# The Myriad Properties of *Pasteurella multocida* Lipopolysaccharide

**DOI:** 10.3390/toxins9080254

**Published:** 2017-08-21

**Authors:** Marina Harper, John Dallas Boyce

**Affiliations:** Infection and Immunity Program, Monash Biomedicine Discovery Institute and Department of Microbiology, Monash University, Clayton, VIC 3800, Australia; marina.harper@monash.edu

**Keywords:** *Pasteurella multocida*, lipopolysaccharide, endotoxin, immunity, virulence

## Abstract

*Pasteurella multocida* is a heterogeneous species that is a primary pathogen of many different vertebrates. This Gram-negative bacterium can cause a range of diseases, including fowl cholera in birds, haemorrhagic septicaemia in ungulates, atrophic rhinitis in swine, and lower respiratory tract infections in cattle and pigs. One of the primary virulence factors of *P. multocida* is lipopolysaccharide (LPS). Recent work has shown that this crucial surface molecule shows significant structural variability across different *P. multocida* strains, with many producing LPS structures that are highly similar to the carbohydrate component of host glycoproteins. It is likely that this LPS mimicry of host molecules plays a major role in the survival of *P. multocida* in certain host niches. *P. multocida* LPS also plays a significant role in resisting the action of chicken cathelicidins, and is a strong stimulator of host immune responses. The inflammatory response to the endotoxic lipid A component is a major contributor to the pathogenesis of certain infections. Recent work has shown that vaccines containing killed bacteria give protection only against other strains with identical, or nearly identical, surface LPS structures. Conversely, live attenuated vaccines give protection that is broadly protective, and their efficacy is independent of LPS structure.

## 1. Diseases Caused by *Pasteurella multocida*

The species *Pasteurella multocida* comprises a heterogeneous set of organisms that are common commensals of the oropharyngeal tract of many vertebrate species [1]. *P. multocida* strains are also the primary causative agent of a wide range of animal diseases, including haemorrhagic septicaemia (HS) in ungulates, fowl cholera (FC) in avian species, atrophic rhinitis (AR) in pigs, and snuffles in rabbits [1]. As well as being primary pathogens, *P. multocida* strains may also be involved as opportunistic pathogens associated with agents of other diseases, including lower respiratory tract infections, such as bovine respiratory disease complex in cattle, and enzootic pneumonia in cattle and pigs. *P. multocida* is also a common cause of bite-associated soft-tissue infections in humans, with 50% of cat and dog bites resulting in wounds contaminated with *P. multocida* [2].

HS is a rapidly fatal disease of ungulates that causes significant economic impact in many African and Asian countries [3]. Infection likely occurs following entry of the organism into tonsillar tissue, and progresses rapidly to a lethal septicaemia. Signs include fever, oedema, respiratory distress, septic shock and widespread haemorrhaging. Once any signs of disease are observed, death is imminent and mortality is nearly 100% [4,5].

FC can manifest as a chronic, acute or peracute disease in most avian species. It causes significant economic impact to poultry industries worldwide, and outbreaks with high mortality are also seen in wild birds, especially waterfowl [1,6]. It is likely that the initial infection with *P. multocida* occurs via the respiratory tract, and, as with HS, may rapidly progress to disseminated disease. Acute and peracute disease involves rapid bacterial multiplication in the liver and/or spleen, and often results in fatal septicaemia. Chronic forms of the disease include localised infections in joints, wattles or nasal sinuses [4,5].

AR in swine results in atrophy and malformation of the nasal turbinate bones. The signs of AR are almost exclusively the result of the action of the *Pasteurella multocida* toxin (PMT). PMT is a 146 KDa protein that is taken up into host cells via receptor-mediated endocytosis, following binding to asialoganglioside surface receptors and positively charged phospholipids [7]. PMT is a potent, anti-apoptotic mitogen. The C-terminal portion of the protein modulates the activity of a range of eukaryotic signalling pathways via activation of G proteins, including Gq, Gi and G12/13, and leads to inhibition of osteoblast differentiation and bone resorption, resulting in the nasal atrophy observed in atrophic rhinitis [7].

## 2. *P. multocida* Virulence Factors

Compared to many other Gram-negative bacteria, the pathogenesis of the various *P. multocida* disease syndromes is poorly understood. While PMT is the critical virulence factor for causation of AR, and its action is now well defined, no exotoxins have been associated with the other *P. multocida* diseases. However, several important virulence factors have been characterised in strains that cause FC and HS disease. The presence of a polysaccharide capsule is a critical virulence factor for FC and HS strains, with defined acapsular mutants being severely attenuated for growth in vivo [8,9]. The filamentous haemagglutinin surface adhesin is also necessary for full virulence in both avian and bovine pneumonia strains [10,11]. The other major *P. multocida* virulence factor that has been well characterised is lipopolysaccharide (LPS). LPS plays a crucial role in pathogenesis, as defined mutants with truncated LPS are less virulent, and more sensitive to killing by host antimicrobial peptides [12,13]. Furthermore, as is the case with the majority of Gram-negative pathogens, *P. multocida* LPS is a primary stimulator of the host immune response and a critical determinant of bacterin protective efficacy [14]. LPS is also an important serological determinant of strain variability and is the primary diagnostic antigen in a number of *P. multocida* typing systems [15,16]. The structure of the *P. multocida* LPS and its role in disease, host immunity, and strain typing, will be the focus of this review.

## 3. General Role of Lipopolysaccharide

The outer leaflet of the outer membrane in almost all Gram-negative bacteria is primarily composed of LPS [17]. As such, LPS plays a crucial role in the interaction between the bacteria and the environment, and is intimately involved in membrane barrier function, bacterial virulence, and host immunity. LPS contains three to four regions; namely, lipid A, which serves as the membrane anchoring component; inner core, which typically contains between one and three 3-deoxy-d-manno-oct-2-ulosonic acid (Kdo) residues, and heptose; outer core, consisting of a number of different sugars and often including glucose, galactose, galactosamine, and glucosamine; and O-antigen (O-polysaccharide), consisting of highly variable and repeating oligosaccharide units [18]. The O-antigen component of LPS is absent in many Gram-negative species, including *Campylobacter jejuni*, *Moraxella catarrhalis*, *Neisseria* spp., and many species within the Pasteurellaceae family, including *Haemophilus influenzae* and *P. multocida.* In these bacteria, the outer core region of the LPS is the most distal and variable part of the LPS molecule. This short or “rough” type of LPS is often referred to as lipooligosaccharide [19]. The LPS of many bacteria can be decorated with a number of different chemical groups, including acyl groups, phosphoethanolamine (PEtn), phosphocholine (PCho) and/or sialic acid [20,21,22].

Lipid A is the endotoxic component of LPS, and strongly stimulates both the innate and adaptive immune response. The lipid A component of LPS acts as a primary pathogen-associated molecular pattern (PAMP) molecule that is recognised by various eukayotic pattern recognition receptors, such as the Toll-like receptors (TLR). The lipid A component of LPS, produced by most Gram-negative bacteria, binds to the plasma protein lipopolysaccharide binding protein (LBP). The CD14 receptor on the surface of phagocytes then binds to LPS/LBP and presents the complex to the LPS binding protein MD2, already associated with the TLR-4 receptor. Following the formation of this complex, oligomerisation of TLR-4 triggers an intracellular signalling cascade and NFκB activation, resulting in the expression of pro-inflammatory cytokines, and recruitment of more immune cells to the site [23,24]. LPS, and in particular, lipid A, is also a powerful trigger for the adaptive immune response. During invasion of the mucosa by Gram-negative bacteria, immature dendritic cells near the site of inflammation (induced by the innate immune response), bind the LPS/LBP complex (via the CD14/MD2 receptor), triggering TLR-4 activation, and the transformation into mature antigen presenting cells that then migrate to the local lymph nodes. A range of antigen-specific T lymphocytes types are then activated, that, in turn, initiate the proliferation of the appropriate B cell populations, and the subsequent production of antibodies specific for LPS and other bacterial components. A cell-mediated immune response may also be activated, depending on the attributes of the invading bacteria, with subsets of CD4^+^ T helper cells recruited to the site of infection [25].

The highly hydrophobic lipid A component of LPS anchors the molecule into the outer leaflet of the outer membrane, and is responsible for inducing endotoxic shock. The general structure of lipid A consists of a di-glucosamine disaccharide, and is highly conserved across most Gram-negative species, though variations in acyl chain number and length, phosphorylation, and glycosylation, occur at both the inter- and intra-species level, and in response to changes in environmental conditions [18]. Importantly, the number of acyl chains in the lipid A molecule, the distribution of acyl chains, and certain substitutions to the head groups, can alter the overall conical shape of the molecule, and this is predicted to interfere with the ability of the LPS to engage the TLR-4 receptor [26]. The structure of the *P. multocida* lipid A molecule has not yet been determined, but it is predicted to have similar endotoxic properties to the lipid A from other Gram-negative bacteria. Indeed, LPS isolated from a *P. multocida* HS isolate was able to induce the clinical signs of endotoxic shock when it was introduced intravenously into buffalo [27]. The polysaccharide components of the LPS (inner and outer core regions), produced by many strains of *P. multocida*, are known, and may include a variety of sugars, including heptose (Hep), rhamnose (Rha), galactose (Gal), glucose (Glc), and amino modified sugars, such as N-acetyl galactosamine (GalNAc) and N-acetyl glucosamine (GlcNAc). The lipid A, Kdo, and the sugars, may also be decorated with PEtn, and in some *P. multocida* strains, the sugars can also be decorated with PCho.

From the early 1970s, strains of *P. multocida* have been differentiated into Heddleston serotypes, using a gel diffusion precipitin test that utilises polyclonal antibodies raised against the heat stable antigens from 16 different *P. multocida* type strains. The 16 standard type strains used in this test were from a wide range of hosts, including cattle, bison, pigs, humans, poultry, and other birds [15,28,29]. Heat stable antigen generated from *P. multocida* is predominantly LPS, and although many isolates are unable to be typed using this system, it was generally accepted that the Heddleston typing system represented 16 distinct LPS molecules [15,28]. Numerous studies have shown that full protection can be provided using inactivated *P. multocida* whole-cell vaccines (bacterins), but these vaccines provided limited or no protection against strains belonging to other Heddleston serotypes, implying that protection is LPS-structure dependent [30]. Until recently, there was no information on the precise structure of the LPS produced by any *P. multocida* strain, so the relationship between Heddleston serotype, LPS structure and host immunity, could not be confirmed. Recent detailed analyses have defined the precise structures of all of the LPS molecules produced by the 16 Heddleston type strains. This knowledge has allowed us to gain an unprecedented understanding of how each LPS molecule is synthesised and the role that particular LPS structures play in virulence and the stimulation of host immunity.

## 4. *P. multocida* Strains Produce Two Different LPS Glycoforms

**Figure 1:**
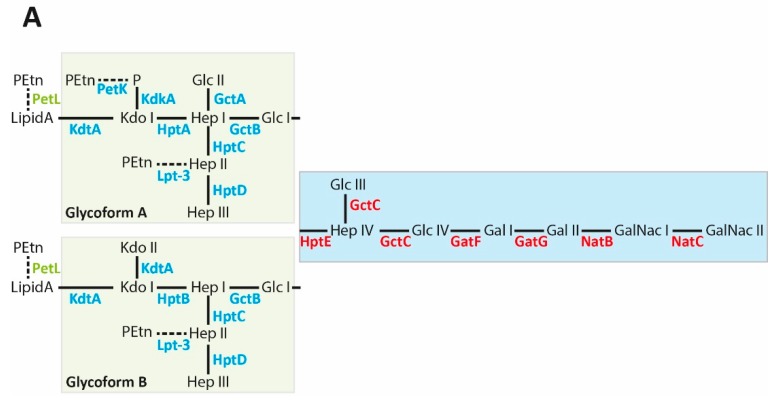

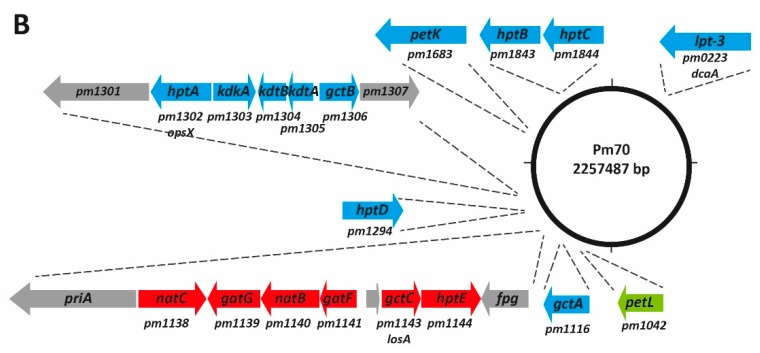
Structure of the lipopolysaccharide (LPS) expressed by *Pasteurella multocida* Pm70, and the genetic organisation of the genes involved in LPS biosynthesis. (**A**) Structure of the Pm70 LPS with the two inner core structures (glycoform A and B) shown at the left (green boxes) and the outer core structure shown at the right (blue box). The enzymes that catalyse each biosynthetic step (inner core biosynthetic enzymes in blue, outer core biosynthetic enzymes in red and the Lipid A PEtn transferase in green) are shown below or beside each of the linkages. Glc, glucose; Hep, heptose; Gal, galactose; GalNAc, *N*-acetyl galactosamine; Kdo, 3-deoxy-d-mannooctulosonate; P, phosphate; PEtn, phosphoethanolamine. Dotted lines show non-stoichiometric additions; (**B**) Genes involved in LPS biosynthesis and their position on the Pm70 circular genome. The outer core biosynthetic locus is shown in red with the non-LPS related genes (*priA*, *rpl31_2* and *fpg*) shown in grey. Inner core biosynthetic enzymes are shown in blue and named according to the current nomenclature. The gene encoding the Lipid A PEtn transferase is shown in green. Arrows designate the direction of transcription of each gene. Where appropriate, previously used gene names/locus tags are shown below the gene.

The structure of the LPS inner and outer core regions has been determined for a large number of strains, including the 16 Heddleston type strains [31,32,33,34,35,36,37,38,39,40]. The inner core region is designated as beginning at the Kdo residue attached to lipid A, and ending at the first glucose (Glc I) residue; the outer core region follows thereafter (Figure 1A). The genes required for inner core assembly are spread across the genome at multiple locations (Figure 1B). Unusually, most *P. multocida* strains produce two inner core structures, designated glycoform A and glycoform B [32,41]. Both glycoform A and B contain Kdo (Kdo I) linked to lipid A, but glycoform B also has a second Kdo (Kdo II) attached to the 4 position on Kdo I [41]. The single Kdo molecule in glycoform A is phosphorylated at the 4 position, and this may also be substituted with a PEtn residue (non-stoichiometric addition) (Figure 1A) [41]. Glycoform A also has a second Glc (Glc II) linked to the 6 position on Hep I. Attached to Kdo I in both glycoforms is a tri-heptose side chain (Hep I–Hep II–Hep III). PEtn may also be attached to the 3 position on Hep II in LPS produced by some strains (Figure 1A) [34]. The last sugar in the inner core region is Glc I, located on the main oligosaccharide chain, and is attached to the 4 position on Hep I.

The synthesis of glycoform A and B is determined by the action of the enzymes KdtA and KdkA [41]. The *P. multocida* KdtA is a bifunctional Kdo transferase that is required for the addition of Kdo I to lipid A, and in glycoform B, the addition of Kdo II to the 4 position on Kdo I. The action of KdtA on the acceptor molecule, lipid A, gives rise to the acceptor molecule lipid A–Kdo I that is utilised by the same enzyme to transfer a second Kdo residue to the 4 position of Kdo I, resulting in a nascent glycoform B molecule. However, the Kdo kinase, KdkA, also utilises the 4 position of Kdo I, and transfers a phosphate to this position. Thus, if KdkA is able to phosphorylate the 4 position on Kdo I before the addition of Kdo II (by KdtA), then glycoform A is produced. Therefore, the relative ratio of glycoform A to glycoform B is likely determined by the relative activity of the KdtA and KdkA enzymes. All strains examined to date produce glycoform A LPS, but in many strains, glycoform B LPS is often only detected at low levels. Therefore, we predict that the Kdo kinase, KdkA, acts at a higher efficiency on Kdo I, compared to the activity of the Kdo transferase, KdtA. The inner core oligosaccharide structures of glycoform A and glycoform B are identical across all the *P. multocida* strains. However, some strains have PEtn on Hep II, and others do not, as a result of a mutation in the gene, *lpt-3*, encoding the PEtn transferase responsible for this addition. These include the type strains representing Heddleston serotype 2 and 5 which can be serologically differentiated even though they produce identical LPS structures except for the presence or absence of PEtn on Hep II. Two monoclonal antibodies raised against purified serotype 2 LPS, that lacks PEtn on Hep II, were unable to recognise the same LPS structure with a PEtn residue attached to the 3 position of Hep II [34].

## 5. The *P. multocida* LPS Outer Core

Structural analyses of the LPS produced by the 16 Heddleston type strains have shown that all express structurally distinct LPSs. *P. multocida* does not produce a repeating O-antigen, so it is not surprising that the majority of LPS structural diversity is found in the outer core sugars and associated linkages. Indeed, there are 15 different outer core structures elaborated by the 16 original strains used to establish the Heddleston typing system [16]. Type strains representing serotype 2 and 5 express an identical outer core structure (see Section 7), but differ in the presence or absence of PEtn on Hep II in the inner core. The transferase and biosynthesis genes involved in the assembly of the different outer core structures have been identified in all of the Heddleston type strains [31,32,33,34,35,36,37,38,39,40]. Directed mutagenesis and complementation/heterologous expression experiments in *P. multocida* have allowed for the identification of the precise genes/enzymes required for the assembly of each sugar onto the nascent LPS molecule produced by a number of strains [16]. In all *P. multocida* strains examined, the genes required for the assembly of the outer core are found between the two conserved, non-LPS biosynthesis related genes, *priA* and *fpg* (Figure 1B) [16]. This strongly suggests that horizontal gene transfer, together with recombination at this specific site, has played an important role in the evolution of the LPS structural diversity observed across the *P. multocida* species.

**Figure 2:**
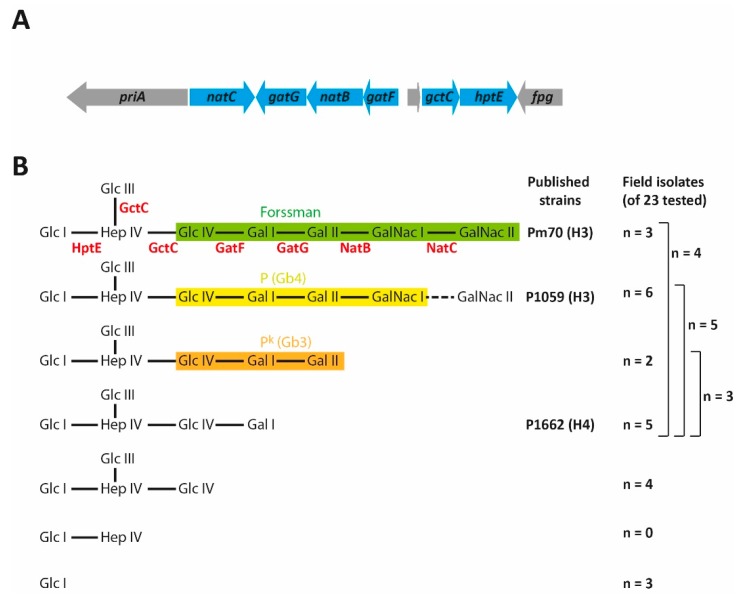
Structures of the LPS expressed by L3 strains. (**A**) Schematic representation of the L3 outer core biosynthetic locus. Arrows designate the direction of transcription of each gene and gene names are shown within the arrow. The non-LPS related genes (*priA, rpl31_2* and *fpg*) are shown in grey; (**B**) all possible LPS structures that could be produced from the L3 outer core biosynthetic locus, and the number of observed structures produced by the Pm70, P1059, and P1662 strains and 23 field isolates. The brackets at the right show the number of isolates that express more than one outer core structure. The enzyme required for each biosynthetic step is shown below or beside each linkage in the full-length LPS structure. The Glc I shown at the far left of each structure is the last sugar of the inner core, and is shown as a point of reference. The dotted line, representing the GalNAcII linkage for strain P1059, indicates that this is a non-stoichiometric addition, and the sugar at this position is only found at very low levels. Regions of the LPS identical to the oligosaccharide components of the Forssman, P, and P^k^ vertebrate glycosphingolipids, are highlighted in green, yellow, and orange, respectively. The Heddleston (H) type of the Pm70, P1059, and P1662 strains are also shown. Glc, glucose; Hep, heptose; Gal, galactose; GalNAc, N-acetyl galactosamine.

Although there are 15 different outer core structures elaborated by the 16 Heddleston type strains, there are only eight unique LPS outer core biosynthesis loci, L1 (includes Heddleston 1 and 14 type strains X-73 and P2225, respectively), L2 (Heddleston 2 and 5 type strains M1404 and P1702, respectively), L3 (Heddleston 3 and 4 type strains P1059 and P1662, respectively), L4 (Heddleston 6 and 7 type strains P2192 and P1997, respectively), L5 (Heddleston serotype 9 type strain P2095), L6 (Heddleston 10, 11, 12, and 15 type strains P2100, P903, P2237, and P1573, respectively), L7 (Heddleston 8 and 13 type strains P1581 and P1591, respectively) and L8 (Heddleston 16 type strain P2723) [16]. LPS compositional analyses on a large number of other *P. multocida* strains has shown that LPS structural diversity is much greater than the 15 structures identified in the Heddleston typing set. This diversity is primarily due to truncations in the LPS outer core as a result of inactivating mutations within individual biosynthesis/transferase genes in the LPS outer core locus. However, mutations may also lead to changes in the transferase donor molecule specificity [39], or to reduced transferase activity or expression [37]. Indeed, only eight of the original Heddleston serovar type strains contain an LPS outer core assembly locus with a full cohort of intact genes, the remaining seven contain nonsense mutations in one or more genes within the locus [16]. For example, the Heddleston serovar 3 and 4 type strains P1059 and P1662, and the genome sequenced fowl cholera isolate Pm70, all have the same L3 outer core biosynthetic locus. Pm70 expresses the full-length outer core structure consisting of Hep IV–[Glc III]–Glc IV–Gal I–Gal II–GalNAc I–GalNAc II (Figure 2). In contrast, the P1059 strain LPS only rarely contains the final sugar, GalNac II, while the P1662 strain assembles an inner core with a very truncated outer core consisting of only Hep IV–[Glc III]–Glc IV–Gal I [37]. As mutation in any of the LPS outer core biosynthesis genes can lead to the truncation of the LPS, it follows that many more than 16 LPS structures exist in naturally occurring strains of *P. multocida*. Indeed, LPS analysis of poultry isolates belonging to the LPS genotype L3 revealed a total of six L3 LPS structures of differing length (Figure 2B) [37]. Thus, the number of viable LPS structures produced by *P. multocida* strains will be determined by the random accumulation of inactivating mutations, with the caveat that particular LPS mutants must be able to survive under the selective pressures encountered in the environment/host. Furthermore, as many of the identified mutations are due to single bp changes, it is likely that some of these mutations are reversible. Thus, the random accumulation of inactivating mutations within the *P. multocida* LPS outer core allows the organism to have some degree of LPS antigenic variability. Interestingly, there is no evidence of phase variable gene expression occurring in *P. multocida*. In *H. influenzae* and other members of the Pasteurellaceae family, variation in LPS structure is achieved primarily through phase variation. This occurs via simple sequence repeats that can expand or contract via slipped-strand mispairing during replication, allowing translation of the protein to occur normally (expression phase on) or be terminated early due to a frameshift mutation (expression phase off) [42].

## 6. The L1 Outer Core Biosynthesis Locus

**Figure 3:**
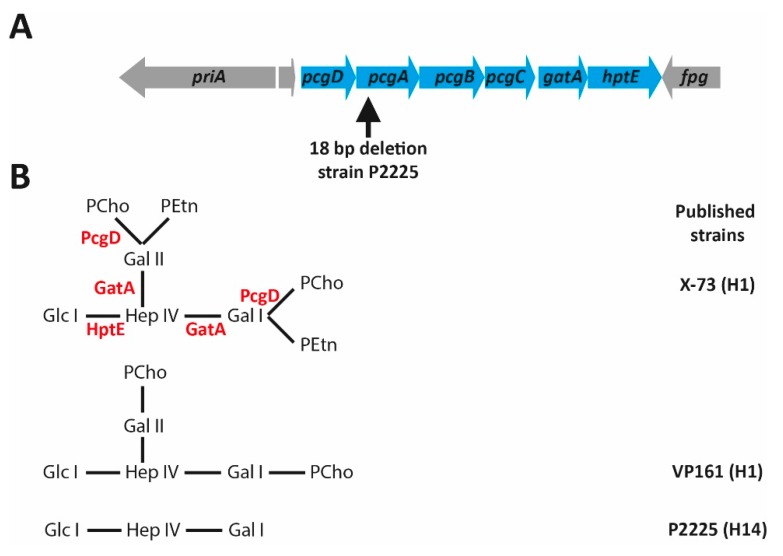

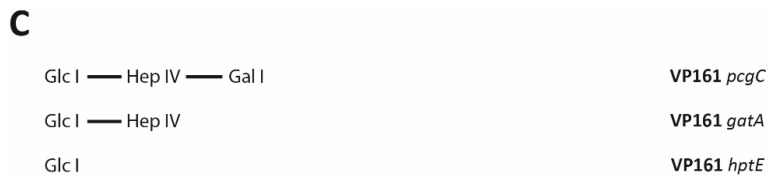
Structures of the LPS expressed by naturally occurring L1 strains and genetically modified VP161 strains. (**A**) Schematic representation of the L1 outer core biosynthetic locus. Horizontal arrows designate the direction of transcription of each gene, and gene names are shown within the arrow. The non-LPS related genes (*priA, rpl31_2* and *fpg*) are shown in grey. The vertical arrow shows the position of the 18 bp deletion observed in the *pcgA* gene of strain P2225; (**B**) LPS structures produced by the field isolates X-73, VP161 and P2225. The enzyme required for each biosynthetic step is shown below, or beside, each linkage in the full-length LPS structure. The Glc I shown at the far left of each structure is the last sugar of the inner core, and is shown as a point of reference. The Heddleston (H) types of the X-73, VP161 and P2225 strains are also shown; (**C**) LPS structures produced by the *pcgC*, *gatA*, and *hptE* VP161 mutants. Glc, glucose; Hep, heptose; Gal, galactose; PCho, phosphocholine; PEtn, phosphoethanolamine.

Strains containing the L1 biosynthetic locus (Heddleston serovars 1 and 14) include many FC isolates, and the well-studied FC strains X-73 and VP161. Strain X-73 produces the most decorated LPS of this group of strains, with an outer core consisting of Hep IV linked to Gal I and Gal II at the 4 and 6 position, respectively. Both Gal residues are decorated with one PCho and one PEtn molecule attached to the 3 and 6 position, respectively [35] (Figure 3). Strain VP161 produces a very similar LPS outer core structure to that elaborated by X-73, except that the VP161 structure lacks the terminal PEtn residues on Gal I and Gal II. This difference is due to an inactivating mutation in the gene encoding the PEtn transferase responsible for this addition (our unpublished data). Inactivation of *gatA* in VP161 results in a truncated LPS outer core consisting of a single heptose (Figure 3C), indicating that the encoded galactosyltransferase, GatA, is bifunctional, and transfers both Gal I and Gal II to Hep IV. However, the Heddleston 14 type strain, P2225, produces a truncated outer core structure consisting of only Hep IV and Gal I. Analysis of the strain P2225 outer core biosynthesis locus identified an 18 bp deletion in *pcgA* that encodes the choline kinase used in the PCho biosynthesis pathway in bacteria [13], indicating that GatA is unable to transfer the second Gal residue to the LPS in the absence of PCho. Similarly, mutation of the *pcgC* gene in VP161, encoding the CTP:phosphocholine cytidylyltransferase, resulted in an outer core structure identical to the P2225 LPS, consisting only of Hep IV and Gal I (Figure 3) [13]. Together, these data show that GatA does not require PCho to add Gal I to Hep IV, but does require PCho on Gal I before addition of Gal II. Thus, the order of assembly in the L1 outer core is predicted to be as follows; Gal I is transferred to the 4 position on Hep IV by GatA, then PCho is transferred to the 3 position on Gal I by the phosphocholine transferase PcgD. This is followed by the PCho-dependent transfer of Gal II by GatA to the 6 position on Hep IV, followed by the addition of the final PCho residue to the 3 position on Gal II by PcgD.

Analysis of 48 *P. multocida* strains isolated from infected avian species identified 11 (23%) that contained the L1 LPS outer core biosynthesis locus [16]. LPS analysis was completed for six of these strains and showed that five elaborated a full-length L1 LPS structure (some with PEtn residues on Gal I and Gal II, and some without these PEtn residues), and one produced a truncated L1 LPS, identical to the LPS produced by Heddleston 14 type strain P2225. While this is a very small sample size, it is likely that strains elaborating a full-length L1 LPS have a selective advantage in avian species. Indeed, all isogenic mutants of strain VP161 that expressed truncated LPS showed significantly reduced in vivo fitness in chickens, and increased sensitivity to chicken antimicrobial peptides, primarily due to the loss of PCho [13,33]. Moreover, chicken infection assays with defined LPS mutants producing an LPS ending in heptose (either Hep I in the inner core or Hep IV in the outer core), showed that these strains were only able to persist at the site of injection, but were not recovered from the blood. Similarly, while L1 LPS structures similar to those produced by the Heddleston 1 and 14 type strains occur in field isolates, none of the L1 LPS glycoforms isolated from natural FC isolates terminate at Hep. Together, these data suggest that *P. multocida* FC mutants producing LPS structures that end with Hep have a strong selective disadvantage in the field. Heptose is commonly produced by bacteria, but is not found in mammals [43], and is rarely detected in higher organisms. It is possible that heptose may act as a “red flag” to the avian host immune system. Recent studies in *Neisseria gonorrhoeae* have identified an intermediate metabolite within the heptose biosynthesis pathway, heptose-1,7-bisphosphate, as a microorganism-associated molecular pattern (MAMP) which is recognised by host cell pattern recognition receptors. Binding of this intermediate to the host immune cell was shown to trigger a signalling cascade that results in cytokine production [44]. However, as yet, there is no evidence that LPS-associated heptose on the surface of Gram-negative bacteria can trigger the same response. Indeed, experiments using an *N. gonorrhoeae* mutant that produced LPS ending with an inner core heptose showed that this LPS molecule failed to induce the same host response that occurred following exposure to heptose-1,7-bisphosphate [44].

## 7. The L2 Outer Core Biosynthesis Locus

Strains containing the L2 LPS outer core biosynthetic locus include the type strains, M1404 and P1702, belonging to the Heddleston serovars 2 and 5, respectively. All strains containing the L2 biosynthetic locus express LPS with an identical outer core structure containing two different heptose isomers. l-*glycero*-d-*manno*-heptose (LD-Hep) is linked to a d-*glycero*-d-*manno* heptose (DD-Hep), followed by GalNAc and Gal [34]. d-*glycero*-d-*manno* heptose is not found in any other LPS structure, and strains belonging to the L2 LPS genotype are the only *P. multocida* strains known to incorporate two different isomers of heptose in their LPS [34,45]. Strains within the L2 genotype can be further divided into two main groups using serology: those with a PEtn on the inner core Hep II (formerly Heddleston serovar 5), and those lacking PEtn on Hep II (formerly Heddleston serovar 2). The addition of PEtn onto Hep II in *P. multocida* is catalysed by the PEtn transferase Lpt-3 [34]. The gene encoding Lpt-3 is functional in P1702, but contains a nonsense mutation in strain M1404. Analysis of the reactivity of selected monoclonal antibodies raised against LPS purified from M1404 (serovar 2 type strain) identified two that reacted strongly to M1404 LPS, but had a significantly reduced binding to P1702 LPS (serotype 5 type strain) [34], showing that the presence of PEtn on Hep II can inhibit the binding of LPS antibodies. Complementation experiments that provided M1402 with an intact *lpt-3* gene *in trans*, resulted in the addition of PEtn to Hep II, and corresponding reduction in the binding of the M1402 LPS-specific monoclonal antibodies [34]. Strains lacking PEtn on the inner core Hep II (i.e., serotyped as Heddleston 2 strains) almost exclusively cause HS in ungulates, and all analysed HS strains are phylogenetically very closely related [46]. Analysis of the genomes of 12 HS strains showed that all contained the identical nonsense mutation observed in the M1404 *lpt-3* (at nucleotide 282) [46], indicating that all currently analysed HS strains are descended from a single progenitor that had a spontaneous mutation within this gene. Interestingly, the addition of the PEtn onto Hep II in the Heddleston 5 type strain P1702 is non-stoichiometric; it is estimated that only 40% of the LPS molecules contain PEtn on Hep II [34]. Therefore, ~60% of LPS molecules expressed by the serovar 5 type strain are not decorated with PEtn, and therefore, are structurally identical to the Heddleston 2 LPS molecule, indicating why some Heddleston typing sera often fail to differentiate these strains (Blackall pers. comm.).

## 8. The L3 Outer Core Biosynthesis Locus

Strains that contain the L3 biosynthetic locus, consisting of six glycosyltransferase genes, include those belonging to the Heddleston 3 and 4 serotypes. Strains belonging to this LPS genotype are the most common causative agents of FC. Indeed, analysis of 48 strains isolated from infected avian species identified 26 (54%) that belonged to the LPS genotype L3 [16]. The first genome-sequenced strain, Pm70, elaborates the longest possible L3 LPS structure based on the complete characterisation of the glycosyltransferase genes within the L3 outer core biosynthetic locus. The outer core of the Pm70 LPS is comprised of two Glc, one Hep, two Gal, and two GalNAc molecules (Hep IV–[Glc III]–Glc IV–Gal I–Gal II–GalNAc I–GalNAc II, Figure 2) [37,47]. The LPS produced by the Heddleston 3 type strain, P1059, is one residue shorter than the Pm70 LPS, and terminates at GalNAc I. However, P1059 contains a functional gene encoding the glycosyltransferase NatC that is responsible for the addition of GalNAc II, and trace amounts of the full-length L3 structure are detected in P1059. It is likely that NatC expression/activity is reduced in P1059, relative to Pm70, and that the majority of LPS molecules are transported to the surface of P1059 cells before the addition of the final sugar [37,47].

A detailed analysis of the LPS produced by 26 L3 field isolates collected from avian species [16] showed that a range of truncated L3 LPS structures occur naturally (Figure 2), and often within the same strain. However, none of the naturally occurring L3 LPS glycoforms terminated at the Hep IV, supporting the hypothesis that LPS structures terminating in Hep may be detrimental for bacterial fitness during infection in birds (see above). The existence of LPS molecules with partially assembled outer cores in the membrane indicate that the truncated nature of these molecules is not detrimental for the bacteria. Indeed, the three longest LPS structures expressed by different L3 strains are identical to the carbohydrate regions in the globo series of vertebrate glycosphingolipids, namely Forssman, P, and P^K^ [37]. These host glycosphingolipids are found in tissue present in most vertebrates, including birds [48], and Forssman antigen is present on the surface of chicken red blood cells [49]. The L3 LPS outer core structures are likely to show very low immunogenicity due to their similarity with these host self-antigens, which suggests that host mimicry is used by *P. multocida* L3 strains to evade the host innate immune system. It is also predicted that strains of *P. multocida* may decorate the terminal ends of the LPS with sialic acid, a molecule incorporated into glycoproteins and gangliosides located on the surface of most host tissues. Sialic acid uptake mutants generated in strains UC6731 and P1059 (both Heddleston 3/LPS genotype L3) were attenuated for growth in vivo [10,50], and studies using radioactively labelled sialic acid showed that P1059 incorporated sialic acid into a product that generated a low molecular band of similar size to LPS in electrophoretic profiles, but the isogenic sialic acid uptake mutant did not [50]. However, no LPS structural studies have been able to unequivocally identify sialic acid on *P. multocida* LPS, although it is possible that the sialic acid is lost during the LPS purification process. Nevertheless, it is tempting to speculate that sialic acid is yet another LPS decoration used by some *P. multocida* strains to allow evasion of the host immune system and/or to increase virulence. Indeed, other pathogenic species within the Pasteurellaceae family are known to decorate their LPS with sialic acid. In particular, the sialation of the LPS produced by non-typeable strains of *H. influenzae* has been shown to be important for resistance to human serum and for the establishment of biofilms, and is required for persistence in vivo [51,52,53].

Within the 26 L3 field isolates analysed, 12 strains simultaneously elaborated multiple LPS glycoforms of differing length (Figure 2B), including structures identical to those produced by the Heddleston 3 and 4 type strains P1059 and P1662, respectively [16]. Not surprisingly, many of these strains react with both the H3 and H4-specific antisera in the Heddleston typing system, and were classified as Heddleston serovar 3:4 [54]. Though the L3 LPS structures mimic host glycosphingolipids, protective antibodies are raised against L3 LPS following vaccination with bacterins containing whole-cell killed L3 strains. Vaccines such as these have been used for decades in the poultry industry, but some have been more effective than others. We predict that bacterin formulations containing a strain that produces multiple L3 LPS glycoforms simultaneously would provide better protection than bacterins containing a strain producing a single L3 LPS glycoform, even if it represents the full-length structure.

## 9. The L4 and L8 Outer Core Biosynthesis Loci

The *P. multocida* L4 biosynthetic locus encodes four glycosyltransferases and an O-acetyltransferase. Strains within the L4 LPS genotype include the Heddleston serovar 6 and 7 type strains, P2192 and P1997, respectively [40], and based on a search of the publicly available genomes, also strain OH1905. The Heddleston serovar 6 type strain produces the full-length L4 LPS structure with an outer core region composed of Gal, GalNAc and 3-*O*-acetylated GalNAc residues (GalNAc3OAc) in the following order; Gal I–GalNAc3OAc I–GalNAc3OAc II–GalNAc–Gal II. The Heddleston serovar 7 type strain P1997 produces a highly truncated LPS structure with an outer core consisting of only a single Gal [40]. Analysis of 48 *P. multocida* avian isolates typed using both Heddleston serotyping and the LPS-specific multiplex PCR, revealed that only four isolates belonged to the LPS genotype L4. Only one of these strains (PM46) was correctly typed as serovar 6 using Heddleston serotyping [16]. PM46 produced a full-length L4 LPS structure, identical to the structure produced by the Heddleston serovar 6 type strain P2192. Of the remaining strains, strain PM1456 was incorrectly serotyped as H14 (LPS genotype 1), and PM1113 and PM1457 could not be typed using the Heddleston system [16]. However, the incorrectly typed strain, PM1456, and the nontypeable strain PM1113, both produced a truncated LPS structure identical to the LPS produced by the Heddleston 7 type strain [16]. Therefore, the Heddleston 7 antisera should have reacted with these strains, highlighting the inaccuracy of the Heddleston serotyping system. The fourth strain belonging to the LPS genotype L4, PM1457, produced two different L4 LPS glycoforms simultaneously; a full-length structure, and one lacking just the terminal Gal (Gal I–GalNAc3OAc I–GalNAc3OAc II–GalNAc) [16]. Thus, as was observed for the L3 strains, it appears that some L4 strains can present LPS molecules that have not been fully extended on the bacterial surface.

The only *P. multocida* strain known to contain the L8 LPS outer core biosynthetic locus is the Heddleston 16 type strain, P2723, that was isolated from a turkey. This strain produces an LPS with a branched-chain outer core structure containing only Gal and GalNAc residues; Gal I–GalNAc I (GalNAc II)–Gal II–Gal III and is similar to the L4 outer core region with respect to the absence of Hep. The L8 locus was likely generated by ancestral recombination events between strains harbouring the L4 and L3 LPS loci, as the locus shows significant nucleotide identity to the L4 locus at one end, and the L3 locus at the other [40].

## 10. The L5 Outer Core Biosynthesis Locus

The *P. multocida* strain P2095 is both the Heddleston serovar 9 type strain and the LPS genotype L5 type strain. Strain P2095 produces a long LPS outer core containing DD-Hep, rhamnose (Rha) and 3-acetamido-3,6-dideoxy-α-D-glucose (Qui3NAc) residues; Hep IV–Rha I–Rha II– QuiNAc I– QuiNAc II–Rha III [36]. This is the only *P. multocida* LPS structure identified to date that contains rhamnose or Qui3NAc [36]. Indeed, LPS containing Qui3NAc has not been identified in any other strains within the Pasteurellaceae family (A.D. Cox pers. comm.). The L5 LPS biosynthetic locus is the largest of all of the LPS outer core biosynthesis loci (13.7 Kbp) and contains all the genes required for the synthesis of rhamnose and Qui3NAc. The type strain P2095 was isolated in the early 1970s from a turkey in the United States of America, and to date, no other field isolates have been identified using the LPS multiplex PCR that contain an L5 LPS locus. Furthermore, none of the publicly available *P. multocida* genomes (representing > 90 strains) contain an L5 LPS biosynthesis locus.

## 11. The L6 Outer Core Biosynthesis Locus

*P. multocida* strains containing the L6 biosynthetic locus include the type strains of the Heddleston serovars, 10, 11, 12, and 15 (P2100, P908, P2237, and P1573, respectively) [39]. The locus contains seven glycosyltransferase genes, but one, *nat_ps*, is a pseudogene in all of the above type strains. The longest L6 LPS structure is produced by the Heddleston 12 type strain, P1573, and contains an outer core consisting of Hep IV–Glc I–Gal I–GlcNAc I–Gal II [39]. The LPS produced by the serovar 15 type strain lacks the last three sugars (Gal I–GlcNAc I–Gal II), and unusually, following the Hep IV, there is a Gal residue replacing the Glc I (Hep IV–Gal). This sugar specificity change is predicted to be the result of a point mutation in the gene *gctD* that changes the donor specificity of the encoded transferase from UDP-glucose to UDP-galactose [39]. The LPS produced by the serovar 11 type strain lacks the last four sugars (Glc–Gal–GlcNAc–Gal) and terminates at Hep IV. All FC strains examined to date produce LPS that does not terminate in a Hep [16], and it is likely that Hep may be detrimental to the fitness of the bacterium in vivo. However, the Heddleston 11 type strain P908 is a swine isolate [55], possibly indicating that with more surveillance, LPS structures terminating with Hep may be detected on isolates from other animal hosts. The LPS produced by the serovar 10 type strain P2100 is the most truncated of all the L6 structures, and terminates at the inner core Glc [39].

The order and linkage of the last four sugars in the full-length L6 LPS outer core (Glc–Gal–GlcNAc–Gal) is identical to the oligosaccharide component of the eukaryotic glycosphingolipid, paragloboside. Such mimicry of eukaryotic glycolipids has also been observed in strains within the LPS genotype, L3, that produce structures identical to the oligosaccharide component of the eukaryotic antigens Forssman, P, and P^K^ (see above). Moreover, mimicry of paragloboside, P1, P, P^K^, and other eukaryotic structures, has been observed in the LPS from other pathogens, including *Haemophilus* spp., *Campylobacter jejuni*, and *Neisseria* spp. [56,57,58].

Of the 48 avian strains analysed during the development of the LPS multiplex PCR [16], seven contained the L6 biosynthetic locus. Four of these strains produced full-length LPS, identical to the LPS produced by the serovar 12 type strain P1573. One isolate produced a fully truncated LPS (no outer core) identical to the LPS elaborated by the serovar 10 type strain P2100, and two strains produced LPS that lacked the last two residues (GlcNAc–Gal). Interestingly, one strain, PM1132, produced two LPS types, one with a composition corresponding to the full-length L6 structure, and one with an LPS outer core composition of three hexoses (Glc or Gal) and one heptose, but no GlcNAc, which does not correspond to any of the known L6 LPS structures. However, genetic analysis of the L6 biosynthetic locus in strain P1573, which produces the longest known L6 structure, revealed two pseudogenes, *nat_ps* and *hetA*, the latter sharing 68% identity with a known galactosyltransferase in the L3 LPS locus, which bioinformatic analyses suggest is ancestrally related [16]. It is possible that the LPS locus in the field isolate PM1132 has retained an intact version of *nat_ps* or *hetA*, allowing for the transfer of a hexose instead of GlcNAc, and thus, an LPS structure that differs from the other observed L6 structures.

## 12. The L7 Outer Core Biosynthesis Locus

*P. multocida* strains containing the L7 biosynthetic locus include the Heddleston 8 and 13 type strains P1581 and P1591, respectively. The P1581 strain produced the longer LPS, with the outer core comprising of one Glc sugar, two Gal sugars, one PEtn (attached to Gal I), one open chain galactosamine residue (1*S*Gal*a*NAc), and an unusual terminal phospho-glycero moiety with the structure 1-((4-aminobutyl)amino)-3-3-hydroxyl-1-oxopropan-2-yl [38]. The LPS produced by the P1591 strain was identical to that produced by P1581, except that it did not contain the terminal phospho-glycero moiety, due to a large deletion within the region of the outer core LPS biosynthesis locus that encoded the genes predicted to be required for the biosynthesis and addition of this molecule [38]. During the development of the LPS multiplex PCR, we identified only a single field isolate (recovered from a Turkey) that had an identical LPS to that produced by P1590 [16,38]. Moreover, none of the publicly available *P. multocida* genomes contain an L7 LPS biosynthesis locus.

## 13. The Role of LPS in *P. multocida* Strain Typing

For the past 40 years, *P. multocida* strains have been typed using a combination of Heddleston serology, which recognises 16 LPS types [15,28,29], and Carter serology, which recognises five different capsular types (A, B, D, E, and F). As described in detail above, while the 16 Heddleston type strains do express structurally distinct LPS molecules, these 16 LPS structures do not cover the full LPS diversity observed in field strains. Indeed, it is possible that different LPS structures can result from the spontaneous mutation of almost any of the LPS biosynthesis genes. This random, low-frequency mechanism of LPS variability is in contrast to the high-frequency, phase-variable expression of LPS biosynthesis genes observed in a number of other bacterial species within the Pasteurellaceae family. Importantly, variation in LPS structure, by whatever means, is an important mechanism to avoid the host immune response [59,60]. Assuming all *P. multocida* LPS mutants retain fitness, this makes the number of possible *P. multocida* LPS structures very large, and therefore, generating a fully discriminatory set of typing sera would be infeasible. Further complicating the accurate use of serologically-based typing is the fact that many strains decorate their surfaces with multiple, but related, LPS glycoforms, each differing in length, some of which may or may not be decorated with PCho and/or PEtn at various sites. Indeed, many L3 strains produce up to four different LPS structures on their surface (Figure 2); including structures identical to those produced by the Heddleston serotype 3 and 4 type strains. It is likely that the typing sera specific for both of these serotypes would react with all these strains, giving rise to the 3:4 serovars reported in the literature. A detailed comparison of the precise LPS structures associated with the Heddleston serotyping scheme has clearly shown that Heddleston serology is unreliable, with the serological typing method correlating with the actual LPS structure at a rate of only 40% [16]. Given the limitations of Heddleston serology, a highly accurate multiplex PCR has been developed to differentiate *P. multocida* strains into 8 different LPS genotypes (L1–L8). This PCR is highly reproducible, and could correctly identify the LPS genotype for 57 of the 58 strains tested [16]. Although the range of LPS structures possible from any known LPS locus can be predicted from the results of the LPS multiplex PCR, it cannot identify the precise structures produced by each strain. This would require mass spectrometry compositional analysis of LPS from each strain, followed by a comparison with the composition of the type strains for which the structure is precisely known. This is currently not feasible for routine typing.

## 14. Role of LPS in *P. multocida* Pathogenesis

As described above, *P. multocida* strains cause a number of diseases in different host species. HS is a disease of ungulates, FC affects avian species, and AR is specific to swine. There is no true correlation between LPS genotype/structure and certain disease types/host predilection, with the exception of HS. This devastating disease of cattle and buffalo is caused exclusively by Heddleston serotype 2 strains belonging to the LPS genotype L2. Genome sequencing and bioinformatic analyses of 12 HS strains revealed that all genomes contained an L2 LPS outer core locus with all four glycosyltransferase genes intact (1 SNP difference across all strains), indicating that all HS strains produced a full-length L2 structure [46]. Analysis also revealed that all HS strains had the same nonsense mutation in *lpt-3*, and they therefore would be unable to modify their LPS inner core Hep II with PEtn. However, the HS strains are phylogenetically very closely related [46] and share many other common properties, so it is not known if LPS type is the primary determinant of host/disease specificity. It would be of interest to generate L2 LPS mutants that produce a truncated L2 outer core, or introduce LPS glycosyltransferase genes that enable the strain to generate a completely different outer core structure, and then determine if these LPS-modified strains could still cause HS. A number of earlier studies have assessed the role of purified LPS in causing the signs of HS. Direct inoculation of semi-purified LPS intravenously in buffalo calves rarely led to death. However, changes in mean rectal temperature, increases in serum TNF, and gross lesions such as generalised congestion, haemorrhage, oedema, and necrosis, were observed in lungs, trachea, heart, liver, spleen, kidney, and nervous tissues [27,61,62]. Thus, inoculation with purified LPS recapitulates some of the signs of HS infection.

A wide range of LPS types are produced by FC isolates. While the majority of FC isolates belong to LPS genotypes L1 and L3 (Heddleston serotypes 1, 3, 4 and 14), strains with L4, L6, and L8 genotypes have also been recovered from avian species. Many FC isolates, especially those belonging to the L3 LPS genotype, only produce truncated LPS structures, or can simultaneously express multiple LPS glycoforms of differing lengths from the same LPS outer core locus. This suggests that full-length LPS is not essential for these strains to cause disease, although it is not known which structures were associated with strains isolated from acute or chronic FC infections. However, for strains within LPS genotype L1, a full-length LPS structure is undoubtedly a critical virulence factor for systemic FC. Directed mutagenesis of strain VP161 showed that a *pcgC* mutant, which produces an LPS outer core containing just Hep IV and a single terminal Gal residue (Figure 3C), was highly attenuated in chickens, compared to the wild type strain, following both intramuscular and intratracheal inoculation (relative competitive index 0.1 and 0.003, respectively) [13]. However, the *pcgC* mutant could still cause lethal disease, albeit with an average time to death of almost double the parent strain. The *pcgC* mutant also showed increased in vitro susceptibility to killing by the chicken antimicrobial peptide fowlicidin-1 (cathelicidin 2); the positively charged PCho residues likely play a role in shielding the bacterial outer membrane from cationic peptide attack [13]. A similar role for PCho in resisting host antimicrobial peptides has been shown for *H. influenza* [63]. *P. multocida* mutants with further truncation of the outer core LPS sugars (*gctB*, *gatA* and *hptE* mutants) had the same level of attenuation as that displayed by the *pcgC* mutant, but a mutation in *hptE*, arresting the transfer of the first outer core sugar (Hep IV) to the LPS inner core, resulted in complete attenuation [33]. The *hptE* mutant, which produced LPS with only the inner core sugars, was also significantly more susceptible to fowlicidin-1 than the *pcgC* mutant [33]. Thus, the LPS outer core is essential for full virulence. Supporting this evidence, LPS compositional analysis has revealed that L1 FC field isolates either have a full-length L1 structure, or one containing two outer core sugars (Hep IV and Gal) [16]. We therefore predict that spontaneous *P. multocida* mutants producing only an LPS inner core would be highly attenuated, and therefore, strongly selected against in natural populations.

As noted above, individual *P. multocida* strains produce two different inner core glycoforms with the same outer core structure attached to each (glycoform A and B, Figure 1). Current data indicate that glycoform A (containing Kdo-P) is always produced in higher abundance than glycoform B (containing Kdo-Kdo) perhaps indicating that glycoform A provides a fitness advantage. However, in *P. multocida* strain VP161, expression of glycoform A is not essential for virulence. A *kdkA* mutant that lacks the ability to phosphorylate the first Kdo, allowing the bifunctional Kdo kinase, KdtA, to add a second Kdo to all nascent LPS molecules has been produced. This mutant produced only glycoform B LPS, and experiments in chickens showed that it was as virulent as the wild type parent strain [41]. Unfortunately, it was not possible to determine if *P. multocida* strains lacking glycoform B LPS are fully virulent, because the addition of the second Kdo to the first Kdo cannot be prevented. This is because the same Kdo kinase is responsible for the addition of the first Kdo (essential for cell viability) and the second Kdo residue. It is currently unknown why most *P. multocida* strains express both glycoform A and glycoform B, although it is possible that each glycoform confers a survival advantage during growth in some, as yet untested, in vivo or environmental niche.

## 15. Role of LPS in Killed Whole-Cell and Live-Attenuated Vaccines

A range of *P. multocida* vaccines have been developed to protect against the different disease syndromes. These include commercial live-attenuated vaccines and killed whole-cell (bacterin) vaccines, as well as farm-specific, “autologous” bacterins [64,65,66]. It has been widely accepted that *P. multocida* bacterins can generate protective immunity that is primarily Heddleston serovar-specific (referred to as homologous protection) [30], while live attenuated strains stimulate some level of protection against a wider group of strains (referred to as heterologous protection) [66]. This suggests that protection is LPS-structure specific for bacterins but this limitation does not apply to live-attenuated strains. However, until recently the lack of knowledge of specific LPS structures has meant that the true coverage of the protection afforded by each vaccine type could not be objectively tested.

**Figure 4:**
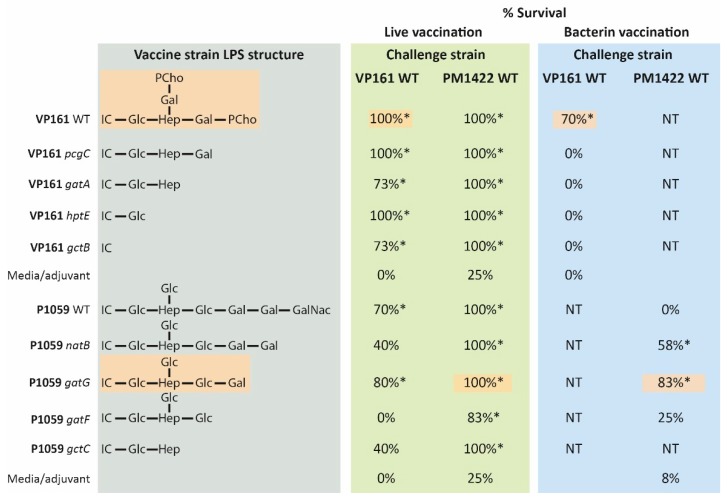
Protective efficacy of live and killed L1 and L3 vaccines against homologous and heterologous challenge in chickens. Live and killed vaccines, each containing an L1 strain (VP161 wild type or a mutant derivative with truncated LPS) or an L3 strain (P1059 wild type or a mutant derivative with truncated LPS), were used to vaccinate chickens using an initial vaccination, followed by a single boost two weeks later. Birds were then challenged two weeks after the booster vaccination, either with the wild type VP161 (L1) strain, or the PM1422 (L3) strain. The vaccine strains and corresponding LPS structures (grey box) are shown at the left. The percent survival following live vaccination is shown in the green box, and percent survival following vaccination with killed whole-cell bacterins is shown in the blue box. The LPS structures of the challenge strains (VP161 and PM1422) are highlighted in orange, as are the corresponding survival percentages for homologous challenge (identical LPS structure). Groups showing significantly increased survival compared to the media only-vaccinated or adjuvant control-vaccinated groups (*p* = 0.05; Fisher’s exact test) are designated by an *. IC, inner core (with the final inner core Glc shown separately for ease of comparison); Glc, glucose; Hep, heptose; Gal, galactose; GalNAc, N-acetyl galactosamine; NT, Not tested. Figure generated using original data from Harper et al. [14].

The precise relationship between LPS structure and protective immunity has been determined using live-attenuated and bacterin fowl cholera vaccines containing the parent strains VP161 (an LPS L1 strain) or P1059 (an LPS L3 strain), and isogenic LPS mutants produced in each parent strain, each of which produces a structurally distinct LPS structure (Figure 4) [14]. All live fowl cholera vaccine strains also contained a mutation in the *aroA* gene to ensure that the strains were avirulent. All bacterin vaccinations provided protective efficacy that was exquisitely sensitive to LPS structure [14]. For example, chickens vaccinated with bacterin containing VP161 (L1 parent strain) were protected against homologous challenge with live VP161 (70%, *p* = 0.003; Figure 4), but chickens that were vaccinated with any of the bacterins containing mutant strains with truncated LPS, such as the VP161 *pcgC* mutant lacking the outer core PCho residues and one Gal, were not protected from the same challenge [14]. Similarly, a bacterin vaccine containing a strain producing the same LPS structure as the challenge strain (L3 P1059 *gatG* mutant), gave solid protection against challenge with the L3 strain PM1422 (83%, *p* = 0.006; Figure 4). Vaccination with a bacterin containing the P1059 *natB* mutant, which produces an L3 LPS structure with one extra Gal compared to the PM1422 challenge strain, also elicited significant, but reduced, protection (58%, *p* = 0.027; Figure 4) [14]. However, bacterins containing any of the other P1059 LPS mutants that produced longer or shorter LPS structures failed to elicit any protective immunity. These data strongly suggest that for FC bacterin vaccines to be effective, they must contain the identical or nearly identical LPS structure/s to those produced by the causative agent of disease. Furthermore, if a virulent strain produces a different LPS structure from that produced by the strain within the bacterin, it is likely that vaccinated birds will not be protected against the virulent isolate. This could easily occur through the introduction of a new strain into the immediate environment or via the generation of spontaneous LPS mutants in *P. multocida* strains already present in chronically infected poultry within a flock.

In stark contrast, the protective immunity conferred by the live attenuated *P. multocida* strains was broad, and not strongly dependent on LPS structure [14]. Indeed, vaccination with any of the strains producing an L1 structure of any length gave significant protection against challenge with the wild type parent, VP161 [14]. Similar results were observed with the L3 live vaccines [14]. Importantly, strong heterologous protection across the two different LPS genotypes, L1 and L3, could also be achieved. Live vaccination with any of the L1 LPS mutants, or the VP161 parent, gave protection against challenge with the L3 strain that produces a completely different LPS structure [14]. However, LPS specificity may play some role in live vaccination, as only two of the L3 LPS mutants elicited significant protection against challenge with the wild type L1 strain VP161, although it is worth commenting that VP161 is highly virulent with an ID_50_ of less than ten bacteria [67]. Overall, it can be concluded that excellent cross-protection can be afforded by live vaccination using a carefully selected vaccine strain, and that, unlike killed whole-cell vaccines, protection is largely independent of LPS structure. It is likely that antibody generation against non-LPS epitopes present on the surface of *P. multocida* is important following live vaccination. It is also possible that cell-mediated immunity is involved in the immune response to live *P. multocida*. It is becoming increasing clear that TH17 helper cells are involved in the recruitment of neutrophils and clearance of extracellular bacteria on mucosal surfaces [68]. Moreover, the activation of this newly identified T helper subset has been shown to contribute to protection against infection with non-typeable *H. influenza* [69]. While it is known that the PMT toxin isolated from *P. multocida* toxigenic strains directly modulates the differentiation of T helper cell subclasses, shifting the response towards Th17 [70], there is only limited data on the role of cell-mediated immunity in response to non-toxigenic strains of *P. multocida* strains. However, a study involving hormonally bursectomised chickens revealed that immunity to *P. multocida* following vaccination could be transferred via intravenous or intraperitoneal transfer of spleen cells to naïve chickens [71].

## 16. Regulation of LPS Biosynthesis Genes

A number of global transcriptional studies have been performed on the *P. multocida* strains X-73 and VP161, as well as a number of defined VP161 mutants. To date, no LPS-associated glycosyltransferase genes or kinase genes have been identified as differentially regulated under any of the conditions examined, indicating that these genes are likely to be constitutively expressed. However, a number of genes involved in LPS modification are likely to be under one or more mechanisms of transcriptional control. Early studies that used DNA microarrays to compare the transcriptomes of in vivo grown *P. multocida* strain X-73 with in vitro grown cells, showed that the non-stoichiometric addition of PEtn to the 4 position on the lipid A, performed by the PEtn transferase PetL (annotated as PM1042 in strain Pm70, our unpublished data), is likely to be transcriptionally controlled. Expression of *petL* was shown to decrease during late-stage in vivo growth, compared to expression in vitro [72]. Interestingly, the expression of another PEtn transferase gene, *lpt-3*, required for the addition of PEtn to Hep II in the inner core [34] of the LPS produced by strain X-73, was simultaneously increased (our unpublished data), raising the possibility that the addition of PEtn to the heptose side chain, rather than to lipid A, provides an advantage during the late stages of systemic infection in chickens. Later studies revealed that inactivation of the sRNA chaperone protein Hfq in the *P. multocida* strain VP161 resulted in an increase in expression of *petL* [73] during mid-exponential phase growth, indicating that the addition of PEtn to lipid A by *petL* is negatively controlled in a Hfq-dependent manner, most likely by an, as yet unidentified, sRNA. The nucleoid-associated protein, Fis, was also shown to be associated with the transcriptional regulation of *petL*. The Fis protein is expressed very highly during early exponential stage growth of *P. multocida* in vitro, but shows reduced expression during late exponential phase growth. Inactivation of the *fis* gene in *P. multocida* strain VP161 resulted in reduced expression of *petL* [74], indicating that the expression of *petL* is positively regulated by the Fis protein. Given that Fis expression is growth-phase dependent, it is likely that *petL* expression is highest when the expression of *fis* is high during the early stages of bacterial growth. This data is further supported by in vivo experiments that show reduced expression of *petL* in strain X-73 during late stage infection (unpublished data). Together these data may hold the key to when PEtn is added to lipid A in *P. multocida*. During the early growth phases, when Fis is abundant, *petL* is expressed strongly, resulting in PEtn decoration of lipid A. During late-exponential growth, the amount of Fis is reduced, and there is a concomitant reduction in the expression of *petL* (unpublished data). Also, it is proposed that an unidentified Hfq-dependent sRNA negatively regulates the expression of *petL* [73], resulting in a decrease in the addition of PEtn to lipid A. However, no in vivo studies have been performed to determine if this correlation also holds true during *P. multocida* infection in the host. In addition to the predicted regulation of these PEtn transferase genes, the genes involved in the biosynthesis and transfer of PCho onto the two galactose residues located at the distal end of the LPS genotype 1, may also be regulated. Inactivation of *hfq* in strain VP161 resulted in the increased expression of *pcgB* and *pcgD* encoding a choline permease and a PCho transferase, respectively, indicating that the addition of PCho to LPS is likely negatively regulated by a Hfq-dependent sRNA [73].

## 17. Concluding Remarks

While we now have a very detailed understanding of the LPS structures produced by different *P. multocida* strains, and how these different structures affect strain virulence and host immunity, there are still many gaps in our knowledge. There is almost nothing known about the primary structure of the lipid A molecule produced by *P. multocida*, and if it differs from other lipid A molecules, with respect to acyl chain length and modifications, with the exception that we know PEtn is often present on the 4′ position of the lipid A [31,32]. We also do not know whether the *P. multocida* lipid A molecule induces inflammatory mediators via the TLR4/MD2 receptor in the same manner as observed for the lipid A of most other bacteria [18]. It is not known why *P. multocida* produces two inner core structures, glycoform A, that has a single phosphorated Kdo, and, in lower abundance, glycoform B, that contains two Kdo residues [32,41]. It is possible that one inner core structure may be more suitable for the initial attachment to the mucosa, and the other for systemic growth in the host. However, analysis of LPS isolated from the blood of chickens during late stage infection with *P. multocida* strain X-73 showed that the range of LPS glycoforms, and relative amounts of glycoform A and B produced, were highly similar to those produced in vitro (our unpublished data). While it is most likely that a single *P. multocida* cell produces both glycoforms simultaneously, it is possible that there may be two populations; one producing glycoform A, and one producing glycoform B LPS. This would provide the opportunity for one population to dominate in a particular niche where it had a specific survival/growth advantage.

It is clear that *P. multocida* produces an LPS molecule with a variable structure at the distal end. This variability provides significant antigenic variation between strains, and likely allows newly infecting strains to avoid host immunity generated in response to previous infections. The outer core region of the LPS also provides the vulnerable LPS core region some protection from antimicrobial attack. In many other Gram-negative bacteria, the LPS also provides resistance to complement-mediated killing, but to date, there is no evidence that the LPS performs this function in *P. multocida*. Finally, there is currently little information on the role that the terminal PEtn moieties play in the biology of this organism. Given their role in other species, it is likely that they provide a masking effect, thus reducing the immune response to LPS. However, this is yet to be established for *P. multocida*. What is clear is that many strains of *P. multocida,* especially FC strains, produce LPS that mimics host structures. Many isolates also produce capsules with structures identical to host glycosaminoglycans (e.g., hyaluronic acid) and it is likely that some strains also decorate their LPS with sialic acid. Thus, millions of years of selective pressure within eukaryotic hosts has led *P. multocida* to evolve multiple surface structures, which together have allowed this bacterium to avoid immune surveillance, and allows for their in vivo survival. Certainly, the evolution of particular LPS structures has been crucial in the rise of *P. multocida* as a highly successful pathogen of a range of species.
